# Accuracy, readability, and understandability of large language models for prostate cancer information to the public

**DOI:** 10.1038/s41391-024-00826-y

**Published:** 2024-05-14

**Authors:** Jacob S. Hershenhouse, Daniel Mokhtar, Michael B. Eppler, Severin Rodler, Lorenzo Storino Ramacciotti, Conner Ganjavi, Brian Hom, Ryan J. Davis, John Tran, Giorgio Ivan Russo, Andrea Cocci, Andre Abreu, Inderbir Gill, Mihir Desai, Giovanni E. Cacciamani

**Affiliations:** 1 USC Institute of Urology and Catherine and Joseph Aresty Department of Urology, Keck School of Medicine, University of Southern California, Los Angeles, CA USA; 2 Artificial Intelligence Center, USC Institute of Urology, University of Southern California, Los Angeles, CA USA; 3 Urology Section, University of Catania, Catania, Italy; 4 Urology Section, University of Florence, Florence, Italy

**Keywords:** Medical research, Prostate cancer

## Abstract

**Background:**

Generative Pretrained Model (GPT) chatbots have gained popularity since the public release of ChatGPT. Studies have evaluated the ability of different GPT models to provide information about medical conditions. To date, no study has assessed the quality of ChatGPT outputs to prostate cancer related questions from both the physician and public perspective while optimizing outputs for patient consumption.

**Methods:**

Nine prostate cancer-related questions, identified through Google Trends (Global), were categorized into diagnosis, treatment, and postoperative follow-up. These questions were processed using ChatGPT 3.5, and the responses were recorded. Subsequently, these responses were re-inputted into ChatGPT to create simplified summaries understandable at a sixth-grade level. Readability of both the original ChatGPT responses and the layperson summaries was evaluated using validated readability tools. A survey was conducted among urology providers (urologists and urologists in training) to rate the original ChatGPT responses for accuracy, completeness, and clarity using a 5-point Likert scale. Furthermore, two independent reviewers evaluated the layperson summaries on correctness trifecta: accuracy, completeness, and decision-making sufficiency. Public assessment of the simplified summaries’ clarity and understandability was carried out through Amazon Mechanical Turk (MTurk). Participants rated the clarity and demonstrated their understanding through a multiple-choice question.

**Results:**

GPT-generated output was deemed correct by 71.7% to 94.3% of raters (36 urologists, 17 urology residents) across 9 scenarios. GPT-generated simplified layperson summaries of this output was rated as accurate in 8 of 9 (88.9%) scenarios and sufficient for a patient to make a decision in 8 of 9 (88.9%) scenarios. Mean readability of layperson summaries was higher than original GPT outputs ([original ChatGPT v. simplified ChatGPT, mean (SD), *p*-value] Flesch Reading Ease: 36.5(9.1) v. 70.2(11.2), <0.0001; Gunning Fog: 15.8(1.7) v. 9.5(2.0), *p* < 0.0001; Flesch Grade Level: 12.8(1.2) v. 7.4(1.7), *p* < 0.0001; Coleman Liau: 13.7(2.1) v. 8.6(2.4), 0.0002; Smog index: 11.8(1.2) v. 6.7(1.8), <0.0001; Automated Readability Index: 13.1(1.4) v. 7.5(2.1), *p* < 0.0001). MTurk workers (*n* = 514) rated the layperson summaries as correct (89.5–95.7%) and correctly understood the content (63.0–87.4%).

**Conclusion:**

GPT shows promise for correct patient education for prostate cancer-related contents, but the technology is not designed for delivering patients information. Prompting the model to respond with accuracy, completeness, clarity and readability may enhance its utility when used for GPT-powered medical chatbots.

## Introduction

The internet contains a wealth of information, has few barriers to use, and is queried for health information by many users. Therefore, it is a source of information for patients seeking information on prostate cancer. Studies have assessed how the public utilizes search engines, like Google, for looking up health-related information [1–6]. In November of 2022, Chat Generative Pre-trained Transformer (ChatGPT), an internet-based large language model (LLM) chatbot application, was made publicly available [7]. In contrast to Google, in which users input a question and must sift through multiple links for potential answers, ChatGPT is an interactive chatbot in which users input questions and are provided with specific, detailed, and individualized outputs in a chatbot format. By March of 2023, ChatGPT was visited by over one billion monthly users, highlighting its instant popularity and mass-adoption [8].

Given the popularity of this new technology, medical researchers have begun to assess its efficacy in responding to health related inquiries [9]. ChatGPT is known to give inaccurate or false information and its medical information has not been verified for widespread patient consumption [10]. Thus, understanding the quality of ChatGPT generated medical information is crucial, as this technology could potentially be used as a medical chatbot. Prostate cancer is a common diagnosis in older patients that could utilize the internet for health-related questions [11, 12].

Prior research has assessed ChatGPT-based medical information delivery, including within urology [9, 13–15]. Yet, to our knowledge no study has assessed the quality of ChatGPT-generated prostate cancer information from both the urologist and patient perspective, that is surveying both urologists and patients for the quality of ChatGPT outputs. We also attempt to prompt ChatGPT to generate patient-friendlier outputs. We aimed to characterize the accuracy, completeness, and clarity of ChatGPT responses to prostate cancer-specific patient questions from both the urologist and patient perspective to further understand its usability and reliability in prostate cancer care.

## Methods

**Fig. 1:**
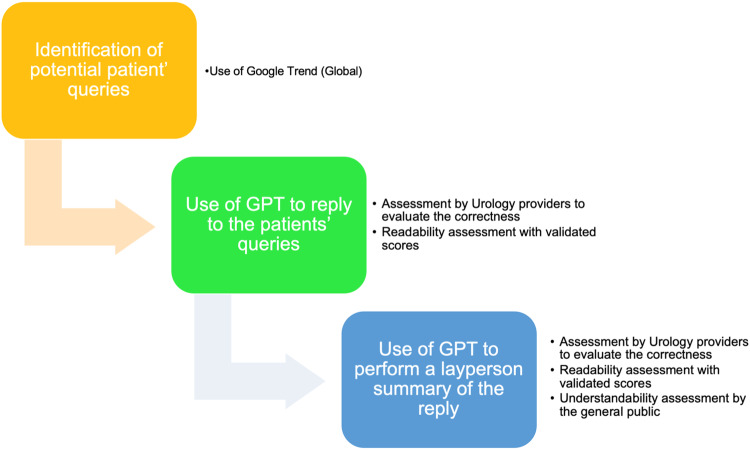
Study Flowchart.

The methods of the present paper rely on a multistep approach (Fig. 1):

### ChatGPT question selection and layperson summary generation

Nine prostate-related questions were developed after searching Google Trends (GT) for the most frequently searched prostate-cancer related questions by the public. Questions were split into three categories: prostate cancer diagnosis, treatment, and postoperative follow-up. Each of the nine questions was inputted into ChatGPT 3.5, with generated outputs recorded for analysis (Supplementary 1). Next, the ChatGPT output was entered into a new ChatGPT window, and the following original prompt was entered to generate a simplified layperson summary: “respond to the above patient question in a way that is understandable at or below a 6th-grade level. Be appropriate, accurate, comprehensive, and clear in the response.”

### Urologist assessment of ChatGPT generated answer

A survey was distributed on RedCap to urological attendings and residents through social media channels and to those who had previously given their consent to be contacted for future research after participating in a survey on the use of GPT in urology, findings of which were published in European Urology [16] available Sep 8-Oct 22, 2023. The survey included the nine ChatGPT-generated responses to prostate cancer-related questions. The survey asked urologists to rate the accuracy, completeness, and clarity of each ChatGPT output. Urologists responded using a five-point Likert Scale for each question (1-strongly disagree, 3-neither agree, nor disagree, 5-strongly agree). Answers receiving a 4 or 5 rating for all three questions were considered to meet the correctness trifecta.

### Urologist assessment of ChatGPT generated layperson summary

Next, two independent reviewers evaluated the simplified ChatGPT outputs intended for laypersons as previously done [14]. Their assessment focused on determining the accuracy of the information provided in these summaries. Additionally, they assessed whether the information was sufficient to enable a patient to make an informed decision. Inter-rate agreement was calculated.

### Readability assessment

The ChatGPT original output and the layperson summary in response to each of the prostate cancer questions were individually inputted into the WebFX readability tool (https://www.webfx.com/tools/read-able/) as previously done [14, 17, 18]. We reported the Flesch Kincaid Reading Ease (FRE), Flesch Kincaid Grade Level (FKG), Gunning Fog Score (GFS), Smog Index (SI), Coleman Liau Index (CLI), and the Automated Readability Index (ARI). For FRE scores, a higher value corresponds with more readable text. For GF, FKG, CL, SMOG, and ARI, a lower value corresponds with more readable text.

### General public assessment of clarity and understandability

The crowdsourcing marketplace Amazon Mechanical Turk (MTurk) (https://www.mturk.com) was utilized to survey the public on the simplified ChatGPT layperson outputs. This tool has previously been used to assess patient opinions on health related information [19, 20]. The survey remained open from Sep 13-Oct 1, 2023. The survey listed the 9 scenarios, and respondents were asked to rate the clarity of the output using a 1-5 point Likert scale. If answered 4 or 5, outputs were deemed clear. Next, MTurk respondent understanding was assessed through a multiple-choice question based on the major theme of the simplified ChatGPT layperson output.

#### Statistical analysis

Mean with standard deviation (SD) and median with interquartile range (IQR) represent continuous variables, while frequencies and percentages (%) represented categorical variables. ANOVA, Chi-Squared (X2), and Fisher exact tests were employed to compare appropriate continuous and categorical variables in univariate analysis. A two-tailed test with *p* < 0.05 was considered statistically significant. The statistical analysis was conducted using SPSS v.24.0 (SPSS Inc. Chicago, IL USA).

## Results

### Assessment of ChatGPT original output and layperson summary

Thirty-six urologists and 17 urology residents assessed the accuracy, completeness and clarity of the original ChatGPT-output for the 9 clinical scenarios on prostate cancer (Table 1). The highest correctness rates were found for scenario 9 with 50 (94.3%) experts rating this GPT-output as accurate, 51 (96.2%) as complete and 51 (96.2%) as clear reaching the correctness trifecta by 48 (90.6%) raters. The lowest approval rate was seen scenario 3 with 38 (71.7%) rating it as accurate, 42 (79.2%) as complete and 47 (88.7%) as clear. The analysis of agreement demonstrated an inter-rater agreement ranging from 88.9% to 100% across the evaluated categories. Two reviewers independently assessing the simplified ChatGPT layperson summaries agreed for 8/9 (88.9%) that the layperson summary provide accurate information, and for 8/9 (88.9%) that the information provided in the layperson summary was sufficient for the patient to make a decision. Details are reported in Supplementary 1.

**Table 1:** ChatGPT output quality rating.

		ChatGPT Output, *n* (%)			
		Accuracy	Completeness	Clarity	Correctness Trifecta
Diagnosis	Scenario 1	51 (96.2)	50 (94.3)	51 (96.2)	46 (86.8)
	Scenario 2	41 (77.4)	47 (88.7)	44 (83.0)	34 (64.2)
	Scenario 3	38 (71.7)	42 (79.2)	47 (88.7)	33 (62.3)
Treatment	Scenario 4	41 (77.4)	41 (77.4)	40 (75.5)	34 (64.2)
	Scenario 5	48 (90.6)	51 (96.2)	49 (92.5)	47 (88.7)
	Scenario 6	46 (86.8)	49 (92.5)	49 (92.5)	46 (86.8)
Follow-up	Scenario 7	48 (90.6)	47 (88.7)	48 (90.6)	45 (84.9)
	Scenario 8	50 (94.3)	51 (96.2)	50 (94.3)	46 (86.8)
	Scenario 9	50 (94.3)	51 (96.2)	51 (96.2)	48 (90.6)

### Readability assessment

**Fig. 2:**
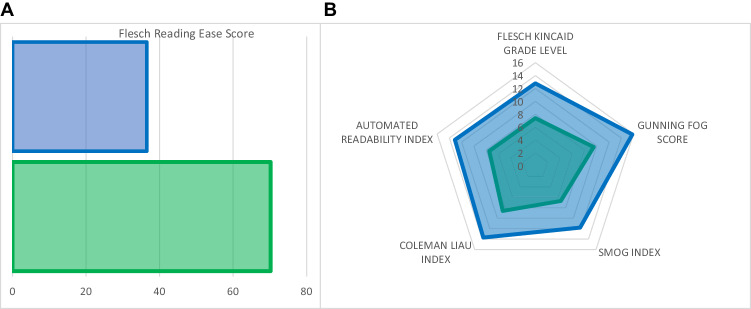
Readability metrics for original ChatGPT output (blue) and layperson summary (green) (*p* < 0.001 for all comparisons). For Flesch Reading Ease Score, high score represents more readable text (**A**); for all other metrics, lower score represents more readable text (**B**).

A summary of the readability scores is provided in Fig. 2. Mean readability of layperson summaries was higher than original GPT outputs ([original ChatGPT v. simplified ChatGPT, mean(SD), p-value] FRE: 36.5(9.1) v. 70.2(11.2), <0.0001; GF: 15.8(1.7) v. 9.5(2.0), *p* < 0.0001; FKG: 12.8(1.2) v. 7.4(1.7), *p* < 0.0001; CL: 13.7(2.1) v. 8.6(2.4), 0.0002; SMOG: 11.8(1.2) v. 6.7(1.8), <0.0001; ARI: 13.1(1.4) v. 7.5(2.1), *p* < 0.0001).

### General public assessment of layperson summaries

514 randomly assigned MTurk workers assessed the clarity and understandability of the layperson summaries for 9 clinical scenarios. The highest approval rate for clarity was seen for the layperson summary for scenario 8 with 492 (95.7%) MTurk workers rating the scenario as clear. Scenario 3 revealed the lowest rate with 460 MTurk workers (89.5%) rating the layperson summary as clear. 449 (87.4%) MTurk workers correctly understood the content of the layperson summary of scenario 2 whereas only 324 (63.0%) correctly understood the content of the layperson summary of scenario 1. Findings are reported in Table 2.

**Table 2:** Layperson summary clarity and understandability.

		Layperson summary, *n* (%)	
		Clarity (Likert Scale 4 & 5)	Understandability
Diagnosis	Scenario 1	482 (93.8)	324 (63.0)
	Scenario 2	486 (94.6)	449 (87.4)
	Scenario 3	460 (89.5)	447 (87.0)
Treatment	Scenario 4	484 (94.2)	436 (84.8)
	Scenario 5	465 (90.5)	432 (84.0)
	Scenario 6	483 (94.0)	418 (81.3)
Follow-up	Scenario 7	477 (92.8)	439 (85.4)
	Scenario 8	492 (95.7)	425 (82.7)
	Scenario 9	479 (93.2)	430 (83.7)

## Discussion

The present study evaluated LLM response quality to relevant PCa queries concerning diagnosis, treatment, and follow-up. It uniquely assessed the output from both patient and provider viewpoints, incorporating objective readability metrics to ascertain if the responses meet the standards required for layperson medical information comprehension. As a quantitative proxy for quality, urologists and urology residents generally rated the original GPT-generated outputs with accuracy, completeness, and clarity. Simplified layperson outputs were generally rated as clear by the public though less demonstrated understandability by correctly answering a multiple-choice question.

This project was initiated after it became apparent to the medical research community that the rapid adoption of LLMs potentially introduces a new source of medical information for patients [21, 22]. Studies have already begun assessing ChatGPT’s capabilities in generating medical information [9, 23]. It is evident from these previous works that ChatGPT has great potential for implementation in the medical field but falls short in characteristic ways. Namely, misinformation generated by LLMs, termed “Artificial Hallucinations,” are exemplified when false and/or misleading citations are outputted and are problematic for medical implementation [24]. Though outside the focus of this particular study, artificial hallucinations represent a major drawback to the medical employment of LLMs, and are therefore worthy of further investigation. Nevertheless, studies are beginning to demonstrate potential LLMs applications across specialties [25, 26], in healthcare training [27], in medical research [28], and in patient education [14].

Within urology, artificial intelligence applications in general and ChatGPT has already been shown to answer questions related to benign, malignant, and emergent conditions with accuracy, though sometimes missing vital information [9, 29]. With various outcomes, ChatGPT has been shown to provide responses in accordance with urologic guidelines [30], aid clinical decision making [31], improve clinician efficiency [32], and respond to patient questions in pediatric urology and men’s health [33, 34]. Other studies have begun assessing the role of large language models specifically for improving patient education and understanding [14, 35–38]. Our findings provide valuable insights into the specific application of patient education, particularly in addressing frequently asked questions about prostate cancer. Additionally the previous study solely examined the quality of the outputs from the urologist’s viewpoint and did not incorporate feedback from lay individuals on how they perceived the provided information.

Implementing a prompt that instructed GPT/LLM to produce a simplified answer to the prostate cancer question successfully improved the objective readability metrics. Future GPT/LLM-powered medical chatbots should focus not just on the accuracy of their information, but also on the method of delivery to the public [39], ensuring clear understanding. A similar outcome was recently demonstrated while attempting to utilize ChatGPT to produce more readable layperson summaries of scientific abstracts [14]. The FKG indicator is a surrogate for grade-level readability, and the original ChatGPT responses were at best written at a post-secondary grade level. This is higher than the 6th grade level, the recommended standard for patient medical education [40]. With an average FKG readability score of 7.4 for the simplified outputs, the prompt used in this study demonstrates a fast and easy way to make medical information more readable for the public.

To create actionable and comparable evaluations of LLM performance in this use case, it is crucial to evaluate the accuracy of ChatGPT outputs through assessments conducted by both physicians and patients [9, 29]. In the context of patient education, urologists assessed the quality of ChatGPT responses with a focus on accuracy, completeness, and clarity. The initial ChatGPT outputs received high ratings in these three areas. However, it is worth noting that unanimous agreement on output quality was not achieved, as ChatGPT does not address all the nuanced medical aspects of each question, which could be better addressed by an expert urologist [41]. This outcome underscores the quality of ChatGPT responses as they pertain to what a typical user from the public might encounter when posing a question to ChatGPT.

A subsequent aim of this study revolved around the potential for ChatGPT to generate contents that are more user-friendly for patients, while maintaining accuracy and necessary information. It is important to acknowledge that this methodology may not precisely mirror real-world patient digital literacy, as future studies are required to understand the users’ role in interpreting AI-generated medical data [42]. Nonetheless, this exercise serves as a valuable step in exploring the capabilities of LLMs in producing medical content that is safe and optimal for patients [38]. Respondents from a crowdsourcing marketplace rated the simplified outputs as clear, although they less frequently exhibited a complete and accurate understanding of the content. This outcome demonstrated that despite scientific concerns that readability metrics of ChatGPT outputs are incompatible with health literacy standards, successful efforts can be made to enhance the information intended for patient consumption.

The results of the present study concur with previous urological studies that LLMs may excel at specific tasks in their current form while performing less well at others [17, 43]. For example, herein we demonstrated less accurate outputs for questions specifically related to PCa diagnosis and treatment compared to follow-up. It is germane to highlight here that OpenAI, the developer of ChatGPT, has made it clear on their website that *“[..] OpenAI’s models are not fine-tuned to provide medical information. You should never use our models to provide diagnostic or treatment services for serious medical conditions* [44].” This cautionary note, coupled with the fact that the study results did not exhibit 100% accuracy in ChatGPT’s outputs, suggests that this technology may not yet be fully reliable for patient education. Further investigations using MedPalm2 [45], specifically designed for medical knowledge, or GPTs trained with specific medical information are needed.

While the transformative potential of this technology is undeniable, its current imperfections must be acknowledged and taken into serious considerations. For the future, we join other researchers in encouraging developers of chatbots intended for medical applications [39] to integrate the most up-to-date medical guidelines and collaborating closely with medical experts to ensure the highest level of accuracy and reliability in responses [38, 46, 47]. It is also worth acknowledging that artificial intelligence outside of LLMs have potential in prostate cancer application and more in general in urology and must also be verified before widespread use [48–51].

This study is subject to several limitations. Firstly, there is a lack of a universally accepted and well-structured tool for evaluating the quality of outputs from LLMs. The inherent stochasticity of this technology could potentially give an output that depends on a given input. Therefore, the findings of this study cannot be generalized to any possible variation of the content of the scenario herein included but just apply to the questions used here as input. Further studies that assess the performance using different versions of the same scenario are awaited. Future research should focus on validating an assessment of chatbot output quality. Secondly, the study utilized version 3.5 of ChatGPT, which is a chatbot that is continuously evolving. Consequently, a replicated study may yield different results as the technology improves over time, due also to the stochastic nature of the GPT outputs to the same input [21]. It is worth noting that this version is currently accessible to the public and likely represents what would be most used publicly at the time this research was undertaken. Third, we did not collect information on the demographics of the MTurk respondents and future studies should utilize stricter survey sampling methodologies. Fourth, we concede the challenges in the non-random allocation of the urology and general population who took the surveys and the resolution of a just basis for volunteer populations, such as urologists, residents, and AMT workers. This may introduce nuanced potential biases in the extrapolation of findings. Lastly, the generalizability of these findings to other LLMs and various cancer types has not been confirmed, highlighting the need for further investigations in this area.

## Conclusions

The present study provides insights into the accuracy and readability of prostate cancer information generated by ChatGPT. This technology shows promise for convenient patient education, though it is not explicitly designed for this purpose. There is also potential to utilize its chatbot interface to produce readable and understandable summaries for the public. Since accuracy is not perfect, better selection of source of information is needed.

## Supplementary information

Supplemental Material 1

## Data Availability

All available in results/supplementary materials.
